# Identification of differentially expressed genes based on antennae RNA-seq analyses in *Culex quinquefasciatus* and *Culex pipiens molestus*

**DOI:** 10.1186/s13071-022-05482-6

**Published:** 2022-10-01

**Authors:** Heting Gao, Zhenyu Gu, Dan Xing, Qiaojiang Yang, Jianhang Li, Xinyu Zhou, Teng Zhao, Chunxiao Li

**Affiliations:** State Key Laboratory of Pathogen and Biosecurity, Beijing Key Laboratory of Vector Borne and Natural Focus Infectious Disease, Beijing, 100071 China

**Keywords:** Mosquitoes, Antennae, Olfactory, Reproduction, Resistance, Vision, RNA-seq

## Abstract

**Background:**

Both *Culex quinquefasciatus* and *Cx. pipiens molestus* are sibling species within *Cx. pipiens* complex. Even though they are hard to distinguish morphologically, they have different physiological behaviors. However, the molecular mechanisms underlying these differences remain poorly understood.

**Methods:**

Transcriptome sequencing was conducted on antennae of two sibling species. The identification of the differentially expressed genes (DEGs) was performed by the software DESeq2. Database for Annotation, Visualization and Integrated Discovery was used to perform GO pathway enrichment analysis. The protein–protein interaction (PPI) network was constructed with Cytoscape software. The hub genes were screened by the CytoHubba plugin and Degree algorithms. The identified genes were verified by quantitative real-time PCR.

**Results:**

Most annotated transcripts (14,687/16,005) were expressed in both sibling species. Among 15 identified odorant-related DEGs, *OBP10* was expressed 17.17 fold higher in *Cx. pipiens molestus* than *Cx. quinquefasciatus*. Eighteen resistance-related DEGs were identified, including 15 from *CYP* gene family and three from acetylcholinesterase, in which *CYP4d1* was 86.59 fold more highly expressed in *C. quinquefasciatus*. Three reproductive DEGs were indentified with the expression from 5.01 to 6.55 fold. Among eight vision-related DEGs, retinoic acid receptor RXR-gamma in *Cx. pipiens molestus* group was more expressed with 214.08 fold. Among the 30 hub genes, there are 10 olfactory-related DEGs, 16 resistance-related DEGs, and four vision-related DEGs, with the highest score hub genes being *OBP lush* (6041148), *CYP4C21* (6044704), and *Rdh12* (6043932). The RT-qPCR results were consistent with the transcriptomic data with the correlation coefficient *R* = 0.78.

**Conclusion:**

The study provided clues that antennae might play special roles in reproduction, drug resistance, and vision, not only the traditional olfactory function. *OBP lush*, *CYP4C21*, and *Rdh12* may be key hints to the potential molecular mechanisms behind the two sibling species' biological differences.

**Graphical Abstract:**

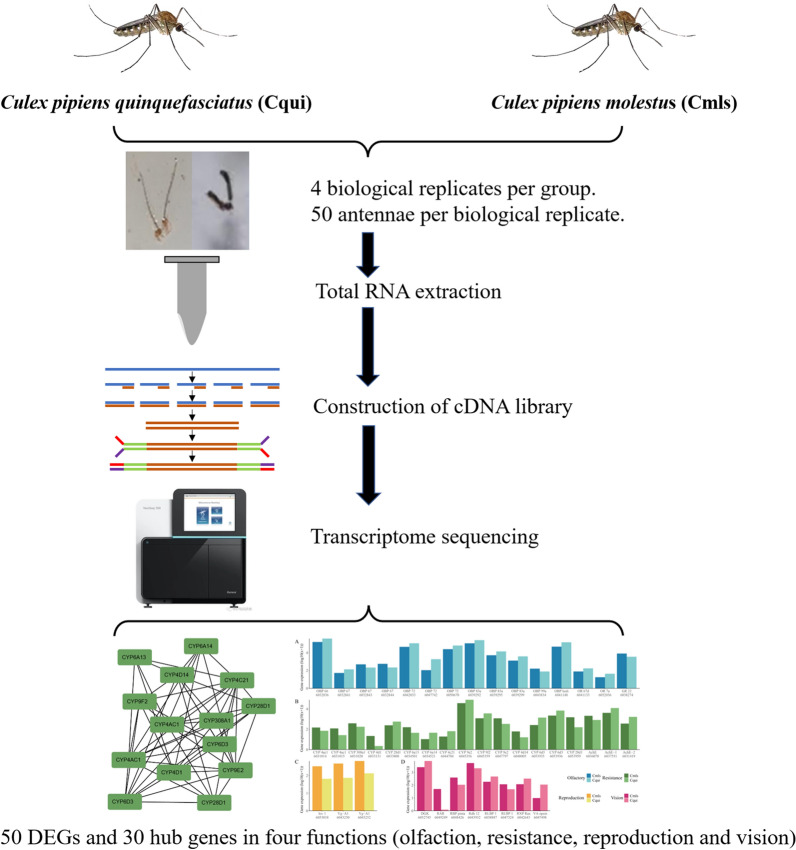

**Supplementary Information:**

The online version contains supplementary material available at 10.1186/s13071-022-05482-6.

## Background

Mosquitoes are not only annoying but can also transmit pathogens, resulting in billions of potential infections and approximately 700,000 deaths worldwide each year. Different species of mosquitoes carry and transmit different pathogens [1, 2]. For example, some species of *Culex* mosquitos are vectors of West Nile virus, Japanese encephalitis virus, and lymphatic filariasis [3].

*Culex quinquefasciatus* (*Cx. quinquefasciatus*, Cqui) and *Cx. pipiens molestus* (*Cx. p. molestus*, Cmls) are sibling species within *Cx. pipiens complex* whose morphologies are difficult to distinguish [4]. However, *Cx. p. molestus* and *Cx. quinquefasciatus* have very different physiological behaviors. For example, *Cx. p. molestus* mainly mate in confined spaces. Their ovaries can develop normally, and they lay eggs without blood-feeding in their first life cycle. On the other hand, *Cx. quinquefasciatus* mate in open areas. There are also differences in their preference for mammalian and bird blood sources [5]. Although the different blood-feeding habits and other physiological behaviors of the two sibling species have been observed for a long time, their possible molecular mechanisms have still not been totally illustrated.

Antenna is the main chemosensory organ of mosquito and plays an important role in smell [6], hearing [7], host-locating [8], and courtship [9]. The mosquito antenna comprises three parts: scape, pedicel, and flagellum. The antenna and maxillary palp detect odors emanating from the host. With the help of proboscis and eyes, which detect taste and visual cues, mosquitoes are successful in flight navigation toward the host [10]. The expressions of many chemoreceptor genes have been found in the antennae of *Anopheles sinensis*, and their expression levels are significantly regulated after the blood meal [11], quite similar to the antennae of the *Culex pipiens* complex [12] and *Aedes aegypti* [13–15]. Besides the typical olfactory function, the antennae of mosquitoes are model systems for other sensations, including acoustics [16].

We conducted transcriptome sequencing on antennae of *Cx. p. molestus* and *Cx. quinquefasciatus* and then explored the differences in the expression and interaction of transcripts. Besides olfaction, antennae could also have special roles in reproduction, drug resistance, and visual function. The study provides hints about the potential molecular mechanisms behind the two sibling species' biological differences.

## Methods

### Mosquitoes

Both *Cx. p. molestus* and *Cx. quinquefasciatus* were obtained from long-term laboratory-reared strains, which had been characterized by male genitalia and/or cytochrome oxidase subunit I (*COI*) barcoding in advance [17, 18]. The breeding conditions were as follows: temperature 26 ± 1 °C, relative humidity 70 ± 5%, and a light:dark regime 14 h:10 h.

### RNA extraction and library construction

Two groups of samples were designed in this study: antennae of *Cx. quinquefasciatus* (Cqui) and antennae of *Cx. p. molestus* (Cmls). Biological samples containing 50 antennae were set in four replicates for each group. RNA was extracted with TRIzol (Takara.9108). Libraries were constructed using the NEB Next Ultra RNA Library Prep Kit and finally sent to Beijing MacroMicro-test Biotechnology Co., Ltd., for transcriptome sequencing.

### Transcriptome sequencing analysis

After the raw data were filtered, clean reads were compared to the reference genome using HISAT2 software [19]. Reference genome file was acquired from the data in the National Center for Biotechnology Information (NCBI)(GCF_015732765.1) [20, 21]. Transcript assembly was performed with StringTie software [22] followed by annotation from databases such as P fam [23], Gene Ontology (GO) [24, 25], and the Kyoto Encyclopedia of Genes and Genomes (KEGG) [26].

A differential analysis was performed using DESeq2 software [27]. The screening criteria for differentially expressed genes (DEGs) in antennae were *padj*  < *0.05 and |log2(foldchange)|*> *1*. Functional annotation and GO enrichment analyses were carried out using the R and clusterProfiler [28, 29] to categorize the DEGs into biological process (BP), molecular function (MF), and cellular components (CC).

Protein-protein interaction (PPI) network analysis obtained the interaction network file through the STRING database [30]. The files were displayed using Cytoscape software [31]. The cytoHubba plug-in Degree topology algorithm was used to predict and explore to calculate gene scores for hub genes [32].

### qPCR verification of identified DEGs

Ten genes with relatively high expressions and significant differences were randomly selected and combined with housekeeping genes 18S ribosomal RNA (*18S*) to verify the accuracy of the transcriptome results. The primers were designed with Oligo Primer Analysis software version 4.0 (Additional file 1: Table S1). A qPCR analysis was conducted with a One Step SYBR PrimeScript RT-PCR Kit II (Cat# RR086A, Takara). The reaction conditions were set as follows: 94 °C for 30 s, 94 °C for 5 s, and 60 °C for 30 s, repeated for 40 cycles. Three technical replicates were performed for each sample. The 2^−ΔΔCT^ method was applied to calculate the relative gene expression [33, 34]. A chi-square test was used to confirm the pairwise differences at the significance level of *α* = 0.05. The correlation between RNA-seq and RT-qPCR expression was calculated by Pearson correlation.

## Result

### Sequencing data quality

After sample data were filtered and the adapter removed, the metagenomic sequencing depth of eight samples was around 30G. The number of valid reads ranged from 22876221 to 31754347. There were 206232097 reads (16,005 annotated transcripts) in total. The Q20 values were all > 95%, suggesting a high degree of quality (Table 1).

**Table 1:** Transcriptome sequencing data mapping summary

Sample	Reads number	Alignment rate (%)	Q20 (%)	GC (%)
Cmls_1	25985588	58.55	97.22	53.16
Cmls_2	24538832	57.97	96.82	52.32
Cmls_3	30471828	60.04	96.1	53.74
Cmls_4	31754347	59.27	95.55	53.86
Cqui_1	23234291	57.02	96.58	53.9
Cqui_2	23513630	57.20	95.1	52.61
Cqui_3	23857360	59.69	95.41	53.01
Cqui_4	22876221	59.47	96.63	54.44

Cmls = antennae of *Cx. p. molestus*; Cqui = antennae of *Cx. quinquefasciatu*. Alignment rate indicated the ratio of sequencing results to reference genome. Q20 = proportion of base mass values  ≥ 20. GC = proportion of GC bases in the sequencing result

### Transcriptome differential expression analysis of antennae

**Fig. 1:**
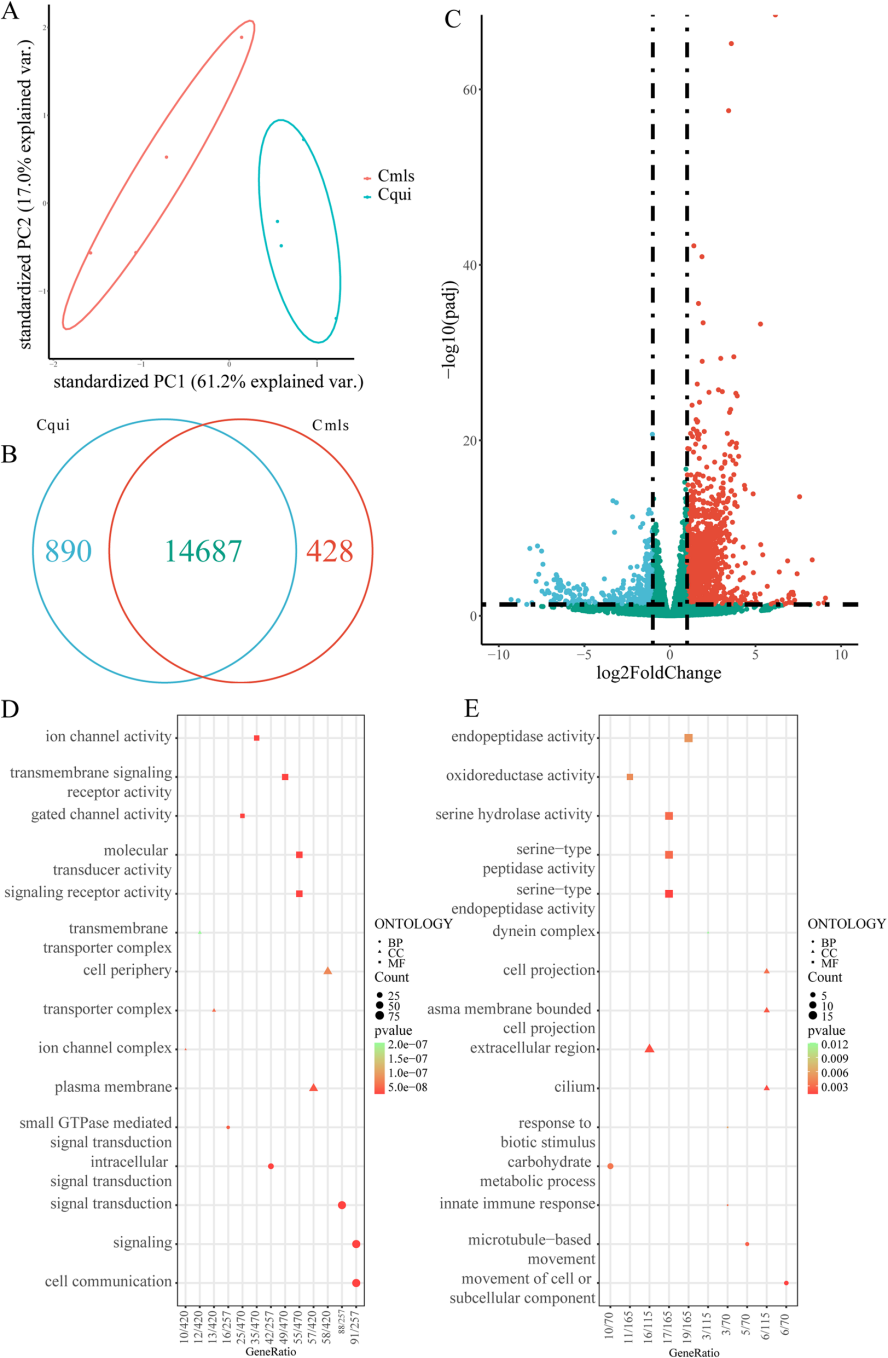
**A** Principal component analysis of the antennal RNA-Seq data of Cqui and Cmls. **B** Venn diagram for two sibling species transcrips. **C** Volcano plot analysis of DEGs between antennal transcriptomes. Green dots indicate DEGs with higher expression in Cqui group, and red dots indicate DEGs with higher expression in Cmls group. **D** GO enrichment analysis of DEGs more expressed in Cqui group. **E** GO enrichment analysis of DEGs more expressed in Cmls group

Principal component analysis showed that the ellipse represented the grouping area with 68% confidence interval; all biological replicates of Cqui and Cmls sample were distributed in two distinct groups (Fig. 1A). Most annotated transcripts (14,687/16,005) were expressed in both sibling species. The number of *Cx. quinquefasciatus*- and *Cx. p. molestus*-specific transcripts were 890 and 428, respectively (Fig. 1B). There were 1577 DEGs in the antennal transcriptome of Cqui and Cmls, of which 1166 DEGs were more highly expressed in the Cqui group and 411 DEGs were higher in the Cmls group (Fig. 1C).

With the purpose of better understanding the differences in regulation between the two sibling mosquito species' antennae, we mapped all transcripts to GO pathways to exploit the pathways that were significantly enriched. DEGs with higher expression in the Cqui were enriched in signal transduction, cell communication in BP; channel complex and neural synapse in CC; signaling receptor activity and channel activity in MF (Fig. 1D). DEGs with higher expression in the Cmls group were enriched in defense response, carbohydrate metabolic process in BP; cell projection, component of endoplasmic reticulum membrane in CC; oxidoreductase activity and ion binding pathways in MF (Fig. 1E).

### Specific functional DEGs in antennal transcriptome

**Fig.2:**
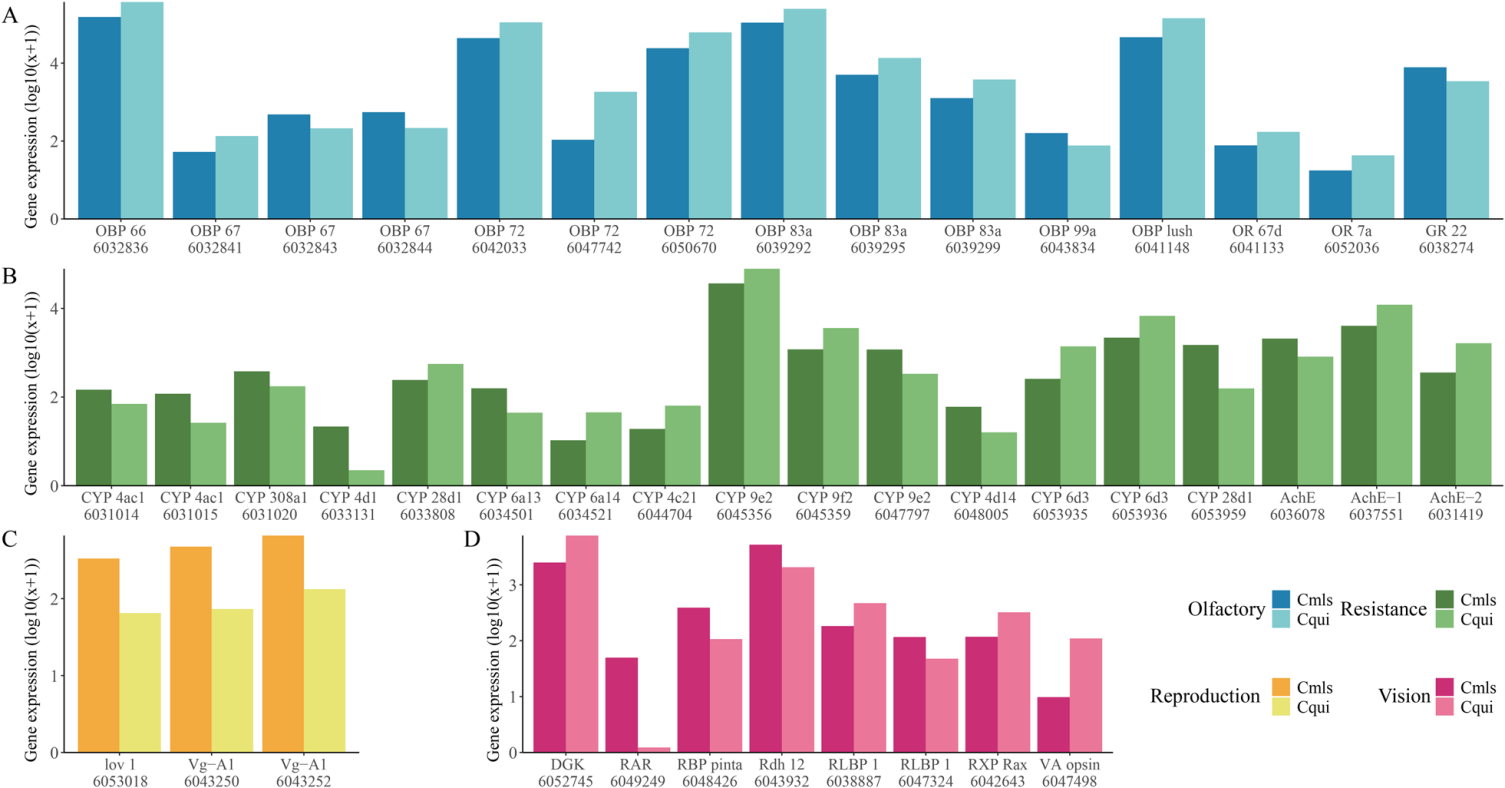
Expression level for specific functional DEGs. Light color-filled columns represent the gene expression of Cqui group; dark color-filled columns represent the gene expression of Cmls group. **A** Expression level of olfactory-related DEGs. **B** Expression level of resistance-related DEGs. **C** Expression level of reproduction-related DEGs. **D** Expression level of visual-related DEGs

By further mining the data, we focused on four types of specific functional DEGs, which would affect the key physiological behaviors of mosquitoes, including olfactory, resistance, reproduction, and vision. A total of 15 odorant-related DEGs were identified, of which 11 were more highly expressed in the Cqui group and four more highly expressed in the Cmls group. Most (12/15) were odorant-binding proteins (*OBP*); two other DEGs (*OR67d* and *OR7a*) were odorant receptors (*OR*) and one (*GR22*) a gustatory receptor (*GR*). Of these, *OBP10* in cmls groups were specially more highly expressed at 17.17 fold times compared with the Cqui group (Fig. 2A, Additional file 1: Table S2).

A total of 18 resistance-related DEGs were identified, including 15 from cytochrome P450 (*CYP*) gene family and three from acetylcholinesterase (*AchE*). Eight *CYP* family DEGs were more expressed in Cqui; for example, *CYP4D1* was 86.59 fold more highly expressed. Three *AchE* genes were differentially expressed with fold change ranging from 0.39–4.59 (Fig. 2B, Additional file 1: Table S3).

Two related vitellogenin-A1 (*Vg-A1*) and one location of vulva defective 1 (*lov-1*) DEGs were more expressed in the Cmls, with expression from 5.01 to 6.55 fold. These three genes were all related to insect mating (Fig. 2C, Additional file 1: Table S4).

Four vision-related genes were more expressed in the Cqui group and four more expressed in the Cmls group. Typically, retinoic acid receptor (*RAR*) RXR-gamma in Cmls group was more expressed at 214.08 fold. Vertebrate ancient (VA) opsin in Cqui group was more expressed with 12.43 fold change (Fig. 2D, Additional file 1: Table S5).

### PPI network for specific functional hub genes

**Fig. 3:**
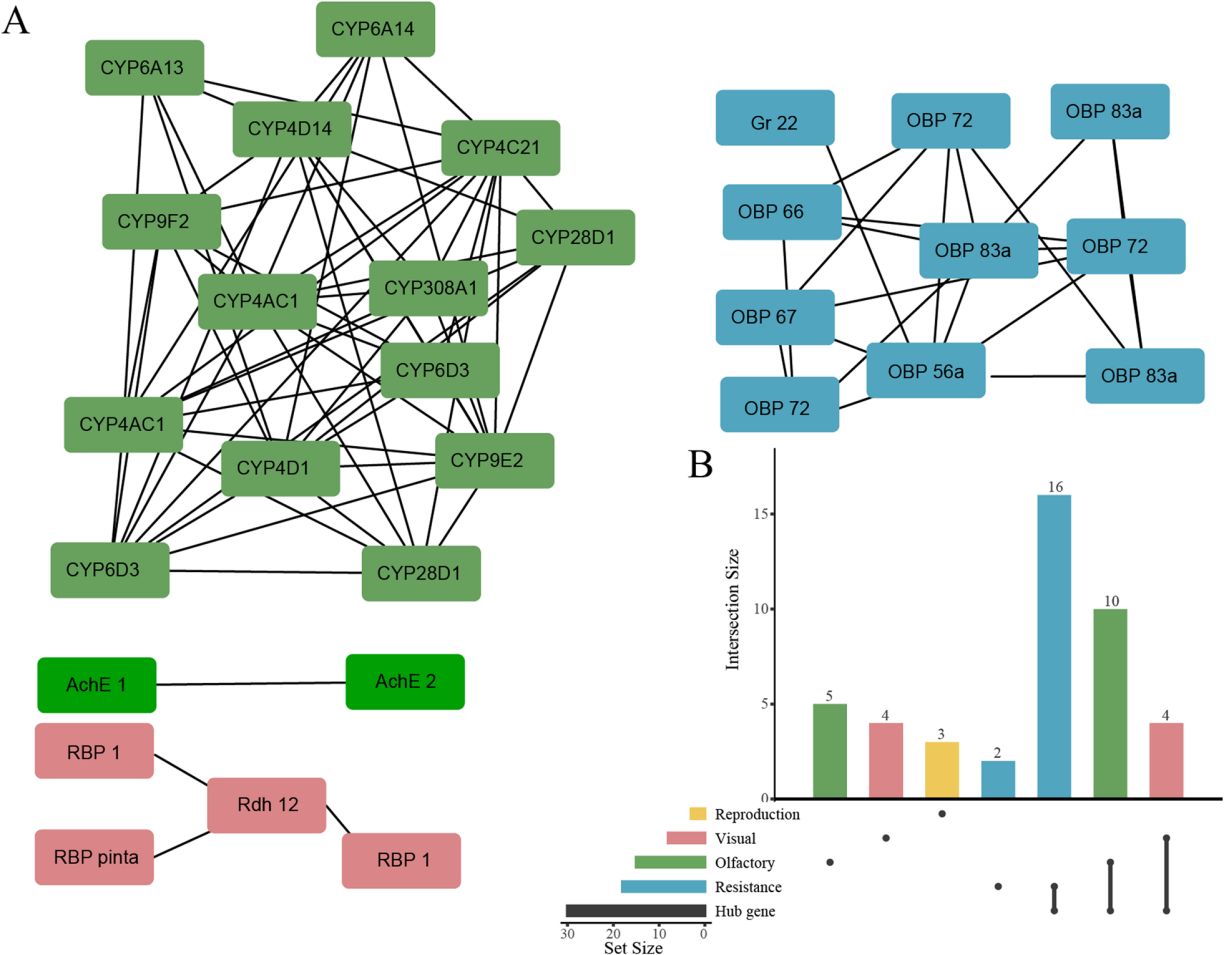
**A** PPI network of top 30 hub genes related to olfaction, resistance, and vision. Green filling represents the resistance-related gene; red filling represents the vision-related gene; blue filling represents the vision-related gene. **B** The upset plot shows the number of each functional gene in the hub gene

Hub genes were the nodes with higher degree, i.e. nodes with more connections in related pathways. With the cytoHubba plug-in and the Degree algorithm, we calculated the hub gene and drew the PPI Networks, PPI (Fig. 3A). PPI was mainly divided into four parts: *CYP* family, olfactory-related genes (*OR*, *OBP*, and *GR*) and *AchE* family and vision-related genes (*RBP* and *Rdh*). The highest scores for specific functional hub genes were *CYP4C21* (6044704), *OBP lush* (6041148), and *Rdh12* (6043932), respectively. Hub genes were closely related internally in each specific function. The hub gene score and function are presented in Additional file 1: Table S6. Among the 30 hub genes, there were ten olfactory-related DEGs, 16 resistance-related DEGs, and four vision-related DEGs (Fig. 3B).

### RT-qPCR validation

**Fig. 4:**
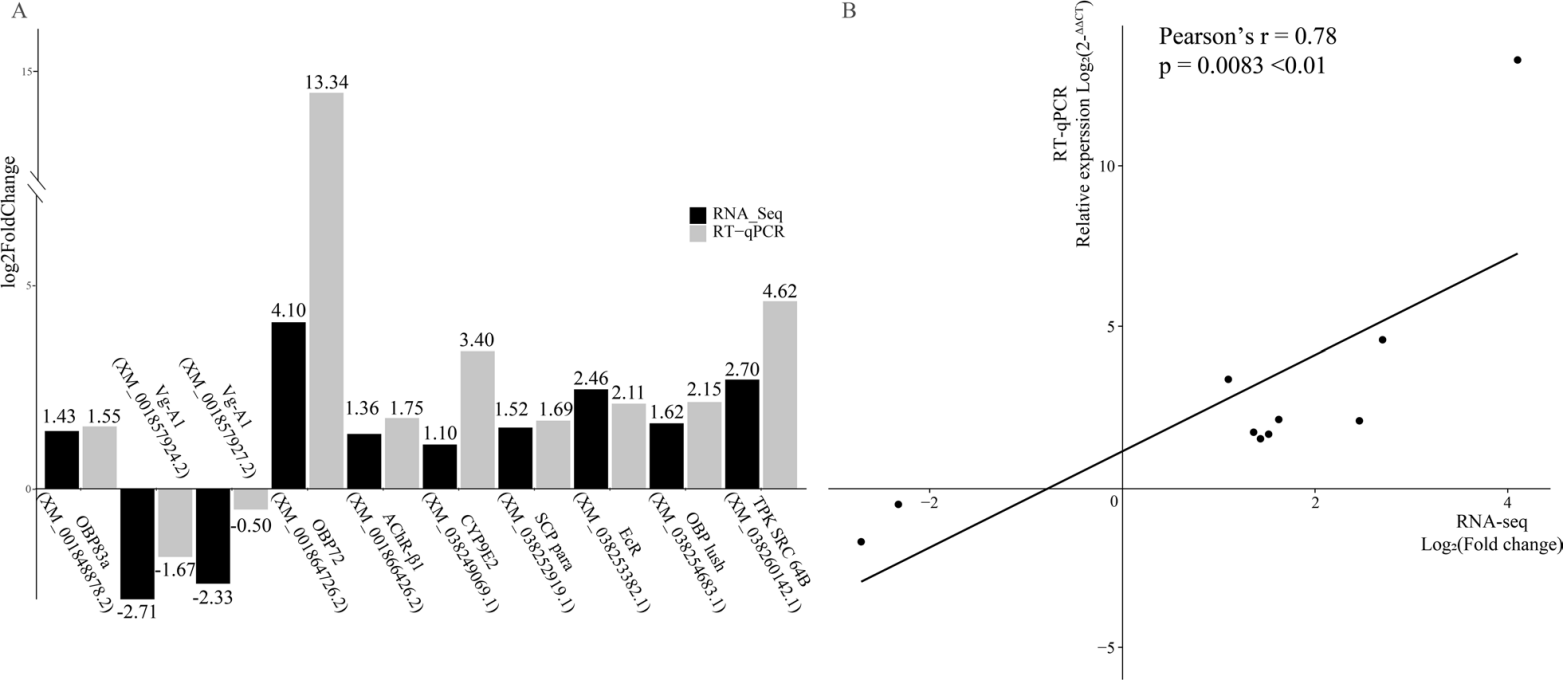
**A** Verification of DEGs in Cqui group and Cmls group using RT-qPCR. Black-filled columns represent the fold change of gene expression based on comparative transcriptome analysis. Gray-filled columns represent data from RT-qPCR analysis. **B** Correlation between RNA-Seq and RT-qPCR results for the tested DEGs using Pearson correlation coefficient (*P* < 0.05)

To validate the reliability of the DEG results, the expression levels of ten selected transcripts were determined by RT-qPCR with the 18S as an internal reference gene. These genes included *OBP*, *Vg*, *CYP*, and so on. All of these genes were significantly different between Cqui and Cmls group on expression of RNA-seq and RT-qPCR (Fig. 4A). The RT-qPCR results were consistent with the transcriptomic data. The correlation coefficient was *R* = 0.78, the significance coefficient *P* = 0.0083 < 0.05 (Fig. 4B).

## Discussion

Most previous studies focused on olfactory genes when conducting transcriptome sequencing on mosquito antennae. For example, 77 *OBPs*, 82 *ORs*, 60 *IRs*, and 30 *GRs* were found in the transcriptome sequencing results of different organs of *A. albopictus* [15]. In the sequencing of the antenna transcriptomes between sibling species *An. coluzzii* and *An. quadriannulatus* [35], *Cx. quinquefasciatus* and *Cx. p. molestus* [12], much olfactory gene expression was significantly regulated to affect host seeking and oviposition.

In this study, 15 odor-related genes were found among the DEGs between *Cx. quinquefasciatus* and *Cx. p. molestus*. Among the olfactory-related DEGs mentioned above, GR22 specifically bound to CO_2_ [36], and OBP66, general OBP83a, OBP6, general OBP72, OBP12, OBP5, and OBP10 were present specifically in the antennae [37, 38]. *Culex pipiens molestus* was more interested in mammals and sometimes birds [39, 40]. The blood-sucking host of *Cx. quinquefasciatus* was more widespread, including birds and other vertebrates [41]. These 15 odor-related genes might reflect the different feeding patterns of the two mosquito species.

There were 16 resistance-related DEGs in the hub gene PPI network, including genes from *CYP* family and *AchE*. *CYP* superfamily catalyzed various modification reactions, such as oxidation, epoxidation, dehydrogenation, hydrolysis, and reduction [42]. Members of *CYP* played critical roles in the detoxification of xenobiotics such drugs, pesticides, and toxins [43]. *AchE* was an effective insecticide target for mosquito vector control [44]. *CYP4AC1* did not overexpress in *Cx. quinquefasciatus* larvae treated with pyrethroids [45]. *AchE1* had been confirmed to be associated with pyrethroid resistance [46]. Higher expression level of *CYP6AA9* was found responsible for deltamethrin-resistant *Cx. pipiens*. [47]. Our study agreed with previous studies confirming the expression levels of resistance-related genes of *Cx. quinquefasciatus* and *Cx. pipiens pallens* were different [48]. The relationship between 16 resistance-related DEGs and resistance needs further verification.

The two *Vg-A1* genes (6043252,6043250) were highly expressed in *Cx. p. molestus*, which may be related to the autogenous habit [49]. In the absence of a blood meal, the ovaries of *Cx. p. molestus* developed normally, which might have led to higher expression of the *Vg* gene.

Of the eight differentially expressed vision-related genes, *RBP1*, *RXP Rax*, eye-specific *DGK*, and VA opsin were more highly expressed in the Cqui group. Retinal oxide-binding protein was abundantly expressed in locust antenna and associated with olfactory-related behaviors in solitary locusts [50]. Eye-specific *DGK* was essential for the photoreceptor function of the *Drosophila* retina [51, 52]. Vertebrate ancient opsin was a green-sensitive photoreceptor that showed high sequence similarity to vertebrate ancient opsin, which might also have affected sexual maturation [53]. *Rdh12* functioned as part of the visual cycle, which was a series of enzymatic reactions required for the regeneration of the visual pigment and detoxification of lipid peroxidation products [54, 55]. Retinol-binding protein transported vitamin A in the hydrophilic environment of the cytoplasm and regenerated visual pigments [56, 57]. *RAR* was involved in the retinoic acid signaling pathway, which was crucial for the control of embryonic development [58]. The different living environments (*Cx. p. molestus* mated mainly in enclosed spaces, while *Cx. quinquefasciatus* mated mainly in open areas) might interact with the differential expression of their vision-related genes.

GO enrichment results showed that genes with higher expression in *Cx. quinquefasciatus* were mainly enriched in cell communication, G protein-coupled receptor signaling pathway, ion channel complex, cell junction, signaling receptor activity, and other pathways. Previous studies have found that G protein-coupled receptor signaling pathway was closely related to insect feeding behavior [59], which affected insecticide resistance in *Cx. quinquefasciatus* by regulating P450-mediated detoxification [60].

Genes with higher expression in *Cx. p. molestus* were mainly enriched in microtubule-based movement, innate immune response, integral and intrinsic component of endoplasmic reticulum membrane synthesis, and cell division-related pathways. Due to the autogenous habit of *Cx. p. molestus*, the body of *Cx. p. molestus* was richer in carbohydrates, lipids, proteins, and other nutrients [49, 61, 62], with vigorous cell proliferation and stronger immune response.

The screened high scored hub genes would be the key to explore mechanisms behind the two sibling species biological differences. *OBP lush* (6041148) had the highest score for olfactory-related hub gene and consisted of a large family of low-molecular-weight, highly divergent proteins expressed exclusively in the chemosensory sensilla of insects. It was required for normal olfactory behavior in *Drosophila* [63]. *OBP lush* mutant flies were abnormally attracted to high concentrations of ethanol, propanol, and butanol but had normal chemosensory responses to other odorants [64].

The resistance-related hub gene *CYP4C21* (6044704) had the highest score, which was 5.4 times higher in the resistant strain compared with the wild *Ae. aegypti* strain in Vietnam [65]. The hub visual correlation gene with the highest score was *Rdh12* (6043932), an NADPH-dependent retinal reductase, which is expressed in the inner segments of the photoreceptors [54]. *Rdh12* could enzymatically reduce toxic lipid 4-hydroxynonenal in vitro [66], protecting cellular macromolecules against oxidative modification and protecting photoreceptors from light-induced apoptosis [67].

Our transcriptome sequencing on antennae of *Cx. quinquefasciatus* and *Cx. p. molestus*, not only focused on olfactory-related genes in common view, but also expanded the orientation of resistance-, reproduction-, and vision-related genes of two sibling species of mosquitoes. Even though the RT-qPCR results confirmed the RNA-Seq prediction to some extent, further molecular and behavioral investigations are needed. This study provides hints about the potential molecular mechanisms behind the two sibling species' biological differences, like blood-feeding, detoxication, mating, host-locating, and other physiological behaviors, which could facilitate the design and development of more targeted repellents or insecticides. By interfering with or silencing the specific genes with RNAi or CRISPR techniques, mosquito host localization ability and resistance to insecticides were affected, leading to the decrease of vector competence. The screened key genes can help to control mosquito-borne diseases effectively and efficiently.

## Supplementary Information

**Additional file 1: Table S1.** The qPCR primers designed to verify RNA-seq results. **Table S2.** Olfactory-related differentially expressed genes. **Table S3.** Resistance-related differentially expressed genes. **Table S4.** Reproduction-related differentially expressed genes. **Table S5.** Visual-related differentially expressed genes. **Table S6.** The hub gene score and function.

## Data Availability

The datasets of this article are included within the manuscript and its supplementary material. All the RNA-seq raw data were submitted to the National Centre for Biotechnology Information (NCBI). Sequence Read Archive with a BioProject ID: PRJNA843865.

## Abbreviations

DEGs: Differentially expressed genes
PPI: Protein–protein interaction
qRT-PCR: Quantitative real-time PCR
Cqui: Antennae of *Culex quinquefasciatus*
Cmls: *Culex pipiens molestus*
NCBI: National Center for Biotechnology Information
GO: Gene Ontology
KEGG: Kyoto Encyclopedia of Genes and Genomes
BP: Biological process
MF: Molecular function
CC: Cellular components
18S: 18S ribosomal RNA
OBP: Odorant-binding protein
OR: Odorant receptor
GR: Gustatory receptor
CYP: Cytochrome P450
AchE: Acetylcholinesterase
Vg: Vitellogenin
lov: Location of vulva defective
RAR: Retinoic acid receptor
VA: Vertebrate ancient
DGK: Eye-specific diacylglycerol kinase
RBP: Retinol-binding protein
Rdh: Retinol dehydrogenase
RLBP: Retinaldehyde-binding protein
RXP: Retinal homeobox protein
AchR: Acetylcholine receptor
SCP: Sodium channel protein
EcR: Ecdysone receptor
TPK: Tyrosine-protein kinase
