# Supplementary material for: Identification of differentially expressed genes based on antennae RNA-seq analyses in Culex quinquefasciatus and Culex pipiens molestus
Source: Parasit Vectors. 2022 Oct 1;15:353. doi: 10.1186/s13071-022-05482-6 (PMC9526932; doi:10.1186/s13071-022-05482-6)
Supplement: Supplementary file 1 — Additional file 1: Table S1. The qPCR primers designed to verify RNA-seq results. Table S2. Olfactory-related differentially expressed genes. Table S3. Resistance-related differentially expressed genes. Table S4. Reproduction-related differentially expressed genes. Table S5. Visual-related differentially expressed genes. Table S6. The hub gene score and function. [file 13071_2022_5482_MOESM1_ESM.docx]

| **NO.** |  | **Gene name** | **Gene description** | **Forward primer** | **Reverse primer** |
| --- | --- | --- | --- | --- | --- |
| 1 |  | CPIJ007608 | general odorant-binding protein 83a | CCACCAGCCTCGCTAATTGA | TCCATGTAGCACTTGAGCCG |
| 2 |  | CPIJ010190 | vitellogenin-A1 | CAGATCGCCAAGGGCTACAA | GCCATTTGAAAAAGTTCCTCACT |
| 3 |  | CPIJ010191 | vitellogenin-A1 | GACCAGAGCGTGAAGCTGTA | GTCCAAATTCATTGCTTTCCGA |
| 4 |  | CPIJ013976 | general odorant-binding protein 72 | AATCAGGCGACATGATGCGA | CGGCGACGATTTTACCCCTA |
| 5 |  | CPIJ016141 | acetylcholine receptor subunit beta-like 1 | TAGAAGATCGCGTGGAGTGC | CGCAGCAAAGTTGTGTCGAA |
| 6 |  | CPIJ012468 | cytochrome P450 9e2 | CGGTGTACATGGTGAAAGATGTTG | TCATCGTTCCAAGTTCCCGT |
| 7 |  | CPIJ017894 | sodium channel protein para | GTTCACTCGACGGAGTCAGA | ACCACAAACGAGTCCTCGAC |
| 8 |  | CPIJ002963 | ecdysone receptor | CGCGTTTGTCTTCGTGTGG | TGACCTTCGATCACTTCGCA |
| 9 |  | CPIJ008793 | general odorant-binding protein lush | CGGTGTGCATAGCCCAAATG | CAGCCTGTGCATCTTTGCAG |
| 10 |  | CPIJ007458 | tyrosine-protein kinase Src64B | TTCTCAGTGTGTGGTGGCTG | TCACCCCAACATAATGTATTCACCT |
| 11 |  | 18S | House-keeping gene | ATTACGTCCCTGCCCTTTGTAC | CACCTTCAAAGACCTCACTAAATAATCC |

Supplmentery table 1. The qPCR primers designed to verify RNA-seq results

Supplmentery table 2 Olfactory-related differentially expressed genes

| **No.** | **Gene ID** | **Preferred Names** | **Names** | **log2FoldChange** | **padj** |
| --- | --- | --- | --- | --- | --- |
| 1 | 6032836 | general odorant-binding protein 66 | odorant-binding protein 58c | 1.280474385 | 5.97E-07 |
| 2 | 6039292 | general odorant-binding protein 83a | odorant-binding protein | 1.171465957 | 0.000303883 |
| 3 | 6041148 | general odorant-binding protein 56a | odorant-binding protein 6 | 1.623507966 | 8.36E-10 |
| 4 | 6042033 | general odorant-binding protein 72 | odorant-binding protein | 1.342457907 | 9.41E-05 |
| 5 | 6050670 | general odorant-binding protein 72 | odorant-binding protein 12 | 1.354874659 | 5.07E-06 |
| 6 | 6039295 | general odorant-binding protein 83a | odorant-binding protein 5 | 1.432589334 | 0.000590227 |
| 7 | 6038274 | gustatory and odorant receptor 22 | gustatory receptor 22 | -1.184271154 | 2.82E-05 |
| 8 | 6039299 | general odorant-binding protein 83a | odorant-binding protein 4 | 1.589008421 | 4.89E-06 |
| 9 | 6047742 | general odorant-binding protein 72 | odorant-binding protein 10 | 4.102177327 | 8.01E-05 |
| 10 | 6032844 | general odorant-binding protein 67 | odorant-binding protein 50d | -1.359860558 | 0.000402479 |
| 11 | 6032843 | general odorant-binding protein 67 | general odorant-binding protein 67 | -1.187748572 | 0.004757913 |
| 12 | 6041133 | odorant receptor 67d | odorant receptor 67d | 1.154108056 | 0.005011264 |
| 13 | 6043834 | general odorant-binding protein 99a | general odorant-binding protein 99a | -1.068405016 | 0.016932451 |
| 14 | 6032841 | general odorant-binding protein 67 | odorant-binding protein | 1.365543613 | 0.001047046 |
| 15 | 6052036 | odorant receptor 7a | odorant receptor 83c | 1.336128514 | 0.039165624 |

Supplmentery table 3 Resistance-related differentially expressed genes

| **No.** | **Gene ID** | **Gene description** | **log2FoldChange** | **padj** |
| --- | --- | --- | --- | --- |
| 1 | 6045356 | cytochrome P450 9e2 | 1.099984315 | 7.91E-05 |
| 2 | 6053936 | cytochrome P450 6d3 | 1.63294518 | 0.034751686 |
| 3 | 6045359 | probable cytochrome P450 9f2 | 1.601204751 | 2.01E-21 |
| 4 | 6053959 | probable cytochrome P450 28d1 | -3.272955488 | 0.00440081 |
| 5 | 6053935 | cytochrome P450 6d3 | 2.43775209 | 2.64E-05 |
| 6 | 6047797 | cytochrome P450 9e2 | -1.827984728 | 0.001088135 |
| 7 | 6033808 | probable cytochrome P450 28d1 | 1.208460201 | 5.62E-06 |
| 8 | 6031020 | probable cytochrome P450 308a1 | -1.124434605 | 8.55E-05 |
| 9 | 6031014 | probable cytochrome P450 4ac1 | -1.08443036 | 0.003985307 |
| 10 | 6034501 | probable cytochrome P450 6a13 | -1.876064334 | 0.038758811 |
| 11 | 6031015 | probable cytochrome P450 4ac1 | -2.269479219 | 0.011631032 |
| 12 | 6044704 | cytochrome P450 4c21 | 1.860317413 | 0.012951893 |
| 13 | 6048005 | probable cytochrome P450 4d14 | -2.057322426 | 0.01746163 |
| 14 | 6034521 | probable cytochrome P450 6a14 | 2.325143162 | 0.020678285 |
| 15 | 6033131 | cytochrome P450 4d1 | -6.436117323 | 0.010303947 |
| 16 | 6037551 | acetylcholinesterase-1 | 1.579009642 | 8.78E-22 |
| 17 | 6036078 | acetylcholinesterase | -1.358549522 | 8.88E-05 |
| 18 | 6031419 | acetylcholinesterase-2 | 2.203062434 | 1.34E-13 |

Supplmentery table 4 Reproduction-related differentially expressed genes

| **No.** | **Gene ID** | **Gene description** | **log2FoldChange** | **padj** |
| --- | --- | --- | --- | --- |
| 1 | 6043252 | vitellogenin-A1 | -2.325334416 | 0.000780582 |
| 2 | 6043250 | vitellogenin-A1 | -2.710400536 | 9.88E-05 |
| 3 | 6053018 | location of vulva defective 1 | -2.373345632 | 0.002171805 |

Supplmentery table 5 Visual-related differentially expressed genes

| **No.** | **Gene ID** | **Gene description** | **log2FoldChange** | **padj** |
| --- | --- | --- | --- | --- |
| 1 | 6038887 | retinaldehyde-binding protein 1 | 1.370925 | 1.57E-05 |
| 2 | 6042643 | retinal homeobox protein Rax | 1.464987 | 3.82E-06 |
| 3 | 6047324 | retinaldehyde-binding protein 1 | -1.30121 | 0.020953 |
| 4 | 6052745 | eye-specific diacylglycerol kinase | 1.603025 | 0.000205 |
| 5 | 6047498 | vertebrate ancient opsin | 3.634519 | 0.001714 |
| 6 | 6043932 | retinol dehydrogenase 12 | -1.34139 | 0.00024 |
| 7 | 6048426 | retinol-binding protein pinta | -1.87737 | 0.014823 |
| 8 | 6049249 | retinoic acid receptor RXR-gamma | -7.74202 | 1.06E-08 |

Supplmentery table 6. The hub gene score and function

| **Rank** | **Gene_name** | **ENTREZID** | **Gene_descriptiom** | **Score** |
| --- | --- | --- | --- | --- |
| 1 | CPIJ011835 | 6044704 | cytochrome P450 4c21 | 11 |
| 1 | CPIJ012468 | 6045356 | cytochrome P450 9e2 | 11 |
| 3 | CPIJ000294 | 6031015 | probable cytochrome P450 4ac1 | 10 |
| 3 | CPIJ000293 | 6031014 | probable cytochrome P450 4ac1 | 10 |
| 5 | CPIJ001758 | 6033131 | cytochrome P450 4d1 | 9 |
| 5 | CPIJ019586 | 6053935 | cytochrome P450 6d3 | 9 |
| 5 | CPIJ014579 | 6048005 | probable cytochrome P450 4d14 | 9 |
| 8 | CPIJ008793 | 6041148 | general odorant-binding protein lush | 7 |
| 8 | CPIJ002537 | 6033808 | probable cytochrome P450 28d1 | 7 |
| 8 | CPIJ012470 | 6045359 | probable cytochrome P450 9f2 | 7 |
| 8 | CPIJ019587 | 6053936 | cytochrome P450 6d3 | 7 |
| 8 | CPIJ019751 | 6053959 | probable cytochrome P450 28d1 | 7 |
| 13 | CPIJ003377 | 6034521 | probable cytochrome P450 6a14 | 6 |
| 13 | CPIJ009568 | 6042033 | general odorant-binding protein 72 | 6 |
| 13 | CPIJ007604 | 6039292 | general odorant-binding protein 83a | 6 |
| 13 | CPIJ000299 | 6031020 | probable cytochrome P450 308a1 | 6 |
| 17 | CPIJ003361 | 6034501 | probable cytochrome P450 6a13 | 5 |
| 17 | CPIJ013976 | 6047742 | general odorant-binding protein 72 | 5 |
| 19 | CPIJ007608 | 6039295 | general odorant-binding protein 83a | 4 |
| 19 | CPIJ002105 | 6032836 | general odorant-binding protein 66 | 4 |
| 19 | CPIJ016949 | 6050670 | general odorant-binding protein 72 | 4 |
| 19 | CPIJ002108 | 6032841 | general odorant-binding protein 67 | 4 |
| 23 | CPIJ011502 | 6043932 | retinol dehydrogenase 12 | 3 |
| 23 | CPIJ007609 | 6039299 | general odorant-binding protein 83a | 3 |
| 25 | CPIJ014674 | 6048426 | retinol-binding protein pinta | 1 |
| 25 | CPIJ013470 | 6047324 | retinaldehyde-binding protein 1 | 1 |
| 25 | CPIJ006622 | 6038274 | gustatory and odorant receptor 22 | 1 |
| 25 | CPIJ007057 | 6038887 | retinaldehyde-binding protein 1 | 1 |
| 25 | Acetylcholinesterase | 6037551 | acetylcholinesterase | 1 |
| 25 | CPIJ000662 | 6031419 | acetylcholinesterase | 1 |
